# The Antidiabetic Mechanisms of Cinnamon Extract: Insights from Network Pharmacology, Gut Microbiota, and Metabolites

**DOI:** 10.3390/cimb47070543

**Published:** 2025-07-12

**Authors:** Rong Wang, Kuan Yang, Xuefeng Liu, Yiye Zhang, Yunmei Chen, Nana Wang, Lili Yu, Shaojing Liu, Yaqi Hu, Bei Qin

**Affiliations:** 1 Xi’an Key Laboratory for Research and Development of Innovative Multi-Target Anti-Hypertensive Drugs, Xi’an Medical University, Xi’an 710021, Chinayangkuan@xiyi.edu.cn (K.Y.); yunmeichen@xiyi.edu.cn (Y.C.); qiqihu1988@xiyi.edu.cn (Y.H.); 2 Xi’an Innovative Anti-Hypertensive Drugs International Science and Technology Cooperation Base, Xi’an Medical University, Xi’an 710021, China; 3 Institute of Drug Research, Xi’an Medical University, Xi’an 710021, China; 4 College of Pharmacy, Xi’an Medical University, Xi’an 710021, China; 18700965583@163.com; 5 Shaanxi Institute of Food and Drug Control, Xi’an 710021, China

**Keywords:** cinnamon, type 2 diabetes mellitus, network pharmacology, gut microbiota, gut microbiota metabolites, intestinal barrier

## Abstract

The progression of type 2 diabetes mellitus (T2DM) is shaped by a multifaceted interplay among genetic, behavioral, and environmental factors, alongside gut dysbiosis. Cinnamon, being abundant in polyphenols and flavonoids, shows significant antioxidant effects. Studies have substantiated that cinnamon contributes to the management of glucose and lipid metabolism. However, the anti-diabetic efficacy of cinnamon is not completely understood. The objective of this research was to clarify the anti-diabetic mechanism associated with cinnamon extract through a combination of chemical profiling, network pharmacology, and in vivo investigations. The results indicated that 32 chemical ingredients, including quercetin, were identified through UPLC-Q-TOF-MS. Network pharmacology revealed that 471 targets related to 14 compounds were screened. The analysis of GO enrichment revealed that the primary pathways were notably enhanced in the metabolism of insulin and glucose. In vivo analyses showed that cinnamon could effectively alleviate hyperglycemia, insulin resistance, and lipid metabolism abnormalities via increased relative abundance of *Akkermansia* and *Ligilactobacillus* at the genus level and a decreased Firmicutes/Bacteroidetes ratio at the phylum level. Moreover, cinnamon reduced the serum levels of lipopolysaccharide (LPS) and proinflammatory cytokines (IL-6 and TNF-α) and significantly increased the colon Zonula occludens-1 (ZO-1) and occludin protein levels. It was also observed that cinnamon improved the fecal SCFA levels (acetic, propionic, butyric, valeric and caproic acid), while also modifying the bile acid (BA) profile and increasing the conjugated-to-unconjugated BA ratio. The Western blotting analysis further demonstrated that cinnamon activated intestinal FXR/FGF15 and hepatic PI3K/AKT signaling pathways. In summary, the finding confirmed that cinnamon ameliorated glucose and lipid metabolism disorders by safeguarding the intestinal barrier and modulating the gut microbiota and metabolites, thereby activating intestinal FXR/FGF15 and hepatic PI3K/AKT signaling pathways.

## 1. Introduction

Type 2 diabetes mellitus (T2DM) is a chronic metabolic disorder characterized by hyperglycemia, insulin resistance, and relative insulin deficiency [1]. T2DM has become a significant global public health concern. Clinically, several oral hypoglycemic agents such as sulfonylureas and biguanides are being used; however, these medications have various adverse effects, such as nausea, vomiting, hypoglycemia, and gastrointestinal discomfort [2,3]. Medicinal herbs are an efficient alternative to modern medicine and can be used to develop anti-diabetic drugs. Given the limitations of existing synthetic drugs, natural products and their active ingredients should be evaluated as potential anti-diabetic agents.

*Cinnamomum cassia* (cinnamon), a member of the *Cinnamomum* genus within the *Lauraceae* family, is widely distributed across the Guangdong and Guangxi regions [4]. In China, cinnamon conforms to the “homology of medicine and food” principle and is rich in aldehydes, polyphenols, and flavonoids [5]. Multiple studies conducted both in vitro and in vivo have demonstrated that cinnamon has anti-oxidative, anti-inflammatory, and immunomodulatory properties [6]. Clinically, cinnamon has been employed to treat various conditions such as arthritis, abdominal pain, diarrhea, and dysmenorrhea [7]. Furthermore, its role in regulating glucose and lipid metabolism has become a research hotspot. In diabetic patients, daily cinnamon has been associated with improved blood glucose and lipid levels [8,9]. A study on a streptozotocin (STZ)-induced diabetic mice model showed that cinnamon polyphenols have hypoglycemic and hypolipidemic effects [10]. However, the mechanism by which cinnamon lowers blood glucose remains elusive. Ethanol is an effective solvent for extracting and recovering phytochemicals from plants. The aim of this study was to evaluate the hypoglycemic effect and mechanism underlying the ethanol extract of cinnamon (EC).

Recently, several studies have indicated the association of the gut microbiota with the onset and progression of various metabolic disorders, including T2DM [11]. The gut microbiome constitutes a vital internal milieu for a healthy organism and plays an indispensable role in maintaining intestinal homeostasis, modulating metabolism, and participating in various important physiological mechanisms of the host [12]. Furthermore, the microbiota produce metabolites, including short-chain fatty acids (SCFAs), bile acids (BAs), and lipopolysaccharide (LPS). which can significantly influence the body’s metabolic and immune functions [13]. An imbalance in microbiota can impair the intestinal barrier, which may contribute to insulin resistance and the development of diabetes [14]. SCFAs and BAs influence lipid and glucose metabolism through the activation of cellular receptors found in tissues such as the pancreas, liver, and intestines [15]. These findings suggest that the gut microbiome and its metabolites may be a potential hypoglycemic target.

The objective of this research was to explore the antidiabetic effect of EC and reveal the role of gut microbiota and metabolites in the pharmacological effects of EC. The research contents included the following steps: (1) delineating the EC components; (2) employing network pharmacology to identify active EC ingredients and their potential targets; (3) investigating the hypoglycemic activity of EC in high-fat diet (HFD) and STZ-induced diabetic mice; (4) identifying the regulatory effects of EC on the gut microbiota, intestinal barrier, and SCFA and BA profiles.

## 2. Materials and Methods

### 2.1. Chemicals and Reagents

The dried cinnamon was sourced from Guangdong, Luoding and identified by Professor Xu-ji Shen from the College of Pharmacy, Xi’an Medical College as the dried bark of *Cinnamomum* cassia Presl (Lauraceae). The specimens were deposited in the herbarium of the College of Pharmacy, Xi’an Medical College (XYXX-2024-016). The reference standards of cinnamic acid (110786-200503) and cinnamaldehyde (110710-201217) were provided by the National Institutes for Food and Drug Control. The HFD (comprising 70% basal chow, 16% sucrose, 12% lard, 1% cholesterol, and 1% sodium cholate) was purchased from Chengdu Dashuo Experimental Animal Co., Ltd. (Chengdu, China). The STZ (No. ACH3380) was supplied by Merck. (Merck, Germany). The metformin (H20023370) was supplied by Merck Pharma (Jiangsu) Co., Ltd. (Nantong, China). The kits for the four lipid parameters were acquired from Nanjing Jiancheng Bioengineering Institute (Nanjing, China). All enzyme-linked immunosorbent assay kits were supplied by Shanghai Yuanju Biological Technology Co., Ltd. (Shanghai, China).

### 2.2. Cinnamon Extract Preparation and Chemical Composition Determination

The cinnamon plant was dried, powdered, passed through a 20-mesh sieve, and extracted using 60% (*v*/*v*) ethanol (plant/ethanol = 1:10, *w*/*v*) for 12 h. Then, ultrasonic extraction was performed thrice for 60 min. The acquired ethanol extracts were combined; evaporated under reduced pressure to obtain a crude ethanol extract; purified using D101 macroporous resin; and eluted with 50%, 70%, and 90% ethanol. The eluates were collected and concentrated under reduced pressure, frozen, and then dried to obtain EC. The extraction rate was 11.41%. The final product was kept at −20 °C in the dark for the subsequent analysis.

The UPLC-ESI-Q/TOF-MS/MS system (Waters Corporation, Milford, CT, USA) was employed to characterize the components in the EC and detect the samples. A Waters BEH C_18_ column (2.1 × 100 mm, 1.8 μm) was utilized for the chromatographic separation at 30 °C. The mobile phase consisted of a mixture of water with 0.1% formic acid and acetonitrile. The elution process occurred as follows: starting with 5% acetonitrile from 0 to 13 min, transitioning to 50–100% acetonitrile between 13 and 30 min, then returning from 100% to 5% acetonitrile during the period of 30 to 32 min and finally maintaining at 5% acetonitrile from 32 to 35 min. The flow rate was consistently maintained at 0.2 mL/min, along with a sample injection volume of 5 μL. Furthermore, the mass spectrometry (MS) spectra were acquired via electrospray ionization (ESI) in positive and negative ion modes with a mass range of *m*/*z* = 50~1000. The parameters for MS detection were as follows: capillary voltage = 2.0 kV; desolvation temperature = 500 °C; desolvation gas flow = 800 L/h; cone gas flow rate = 50 L/h; ion source temperature = 120 °C.

The major compounds, including cinnamic acid and cinnamaldehyde, were analyzed via HPLC (Waters, USA) on a Diamonsil Plus C18-A column (4.6 mm × 250 mm, 5 μm) at 30 °C. The detection wavelength and flow rate were set at 265 nm and 1 mL/min. The mobile phase consisted of a mixture of 0.05% phosphoric acid solution (A) and acetonitrile (B). The gradient elution program was as follows: 0–1 min: 75% A; 1–20 min: 75% to 62% A; 20–30 min: 62% to 60% A; 30–35 min: 60% A; 35–36 min: 75% A; 36–40 min: 75% A.

### 2.3. Network Pharmacological Analysis

The components outlined in Section 2.2 were utilized to determine the active targets of the EC’s ingredients from SwissTargetPrediction (http://www.swisstargetprediction.ch/, accessed on 11 January 2025) and UniProt (https://www.uniprot.org, accessed on 12 January 2025). Furthermore, T2DM-related disease genes were searched in the Gene Cards (http://www.genecards.org/, accessed on 16 January 2025) and OMIM (http://www.omim.org, accessed on 18 January 2025) databases. Duplicate data were removed, then T2DM target genes were screened. Moreover, to identify the overlapping targets between the EC’s active ingredient and T2DM targets, the Venn diagrams were created via Venny 2.1.0 (https://bioinfogp.cnb.csic.es/tools/venny/, accessed on 18 January 2025). The identified genes were then entered into the STRING database (https://cn.string-db.org/, accessed on 20 January 2025) to develop a protein–protein interaction (PPI) network associating EC’s action with T2DM. The network was subsequently brought into Cytoscape 3.9.1 to pinpoint targets with high interactions by applying the Closeness unDir threshold, Betweeness unDir threshold, and Degree unDir threshold criteria. Lastly, the gathered data were uploaded to a web platform (https://www.bioinformatics.com.cn, accessed on 25 January 2025) to conduct enrichment analyses for Gene Ontology (GO) and the Kyoto Encyclopedia of Genes and Genomes (KEGG).

### 2.4. Animals and Treatments

The C57BL/6 mice (4–5 weeks old, 18 ± 2 g body weight (BW), *n* = 48, male) were acquired from the Chengdu Dashuo Experimental Animal Co., Ltd. (Chengdu, China; animal license NO. SCXK (Chuan) 2020-0030). The mice were kept in an environment maintained at 23 ± 3 °C with 50 to 60% relative humidity, on a 12 h light/dark cycle, and given unrestricted access to food and water. After a period of 3 days for acclimatization and adjustment to feeding, eight mice were randomly assigned to the normal control (NC) group, receiving a standard diet. The remaining 40 mice were allocated to the T2DM model group and were fed an HFD. The method for model establishment was based on previous studies [16,17]. After 4 weeks of feeding, each fasted mouse was administered a single intraperitoneal injection of 120 mg/kg STZ (freshly dissolved in 0.1 M citrate buffer with a pH range of 4.2 to 4.5). The NC group received an equivalent dose of the citrate buffer via intraperitoneal injection. The T2MD mice model was confirmed if the fasting blood glucose (FBG) was >11.1 mmol/L after 3 consecutive measurements. Subsequently, the diabetic mice were randomly assigned to five groups (n = 6/group), including the model diabetes group (MD); the 150 mg/kg metformin group (MET); and three EC groups with a low dosage (100 mg/kg; EC-L), medium dosage (200 mg/kg; EC-M), and high dosage (400 mg/kg; EC-H). The MET and EC groups were administered corresponding drugs via gastric gavage once daily for 4 consecutive weeks. While the groups NC and MD were given the same amounts of saline solution, the BW and FBG levels were assessed on a weekly basis. At the end of the trial, all mice were subjected to the oral glucose tolerance test (OGTT) by giving them an oral glucose dose of 2 g/kg, then assessing their blood glucose levels at 0, 30, 60, 120, and 180 min.

### 2.5. Biochemical Analysis of Blood Samples

Before euthanasia, the mice’s blood was collected from their eyeballs and centrifuged at 3000 rpm/min for 10 min to obtain serum, which was then used to assess the TC, TG, LDL-C, and HDL-C levels. The respective ELISA kits(Shanghai Yuanju Biotechnology, Shanghai, China) were utilized to measure the levels of serum LPS, insulin, TNF-α, and IL-6 in each group. Furthermore, the homeostasis model assessment of insulin resistance (HOMA-IR) was assessed: HOMA-IR = FBG × insulin/22.5.

### 2.6. Pathological Examination

The pancreas tissue samples were preserved in 4% paraformaldehyde solution, fixed, washed, dehydrated in gradient alcohol solutions, embedded in paraffin wax, and sliced into 5-μm-thick sections, which were then stained with hematoxylin and eosin (H&E) for the pathological analysis. The sections were observed using a double-blind method and scored for the degree of lesion.

### 2.7. Immunohistochemistry Staining

The tissues from the colon were subjected to fixation, dehydration, and embedding in paraffin, then sliced and incubated with primary antibodies against ZO-1 (1:1500, Servicebio, Wuhan, China) and occludin (1:200, Abcam, Cambridge, UK) overnight at 4 °C. The sections were subsequently rinsed with PBS, probed with HRP-tagged secondary antibodies (1:100, Servicebio, China), and stained with DAB (1:20, ZSGB-BIO, Beijing, China). The counterstaining was performed using hematoxylin. For image acquisition, the BA200 digital microscope (Mike Audi Industrial Group Co., Ltd., Ningbo, China) was employed, and a quantitative analysis was carried out with Halo 101-WL-HALO-1 software (HALO V3.2.1851.439) (Indica Labs, Albuquerque, NM, USA).

### 2.8. Gut Microbiota Assay

On the final day of the experiment, newly obtained fecal samples were aseptically gathered right after defecation and then preserved at −80 °C for the analysis of the gut microbiota. The total DNA was extracted from these fecal samples, and amplification of the V3–V4 region of the bacterial 16S rRNA was performed using PCR. The amplified products were evaluated via gel electrophoresis and quantified via a fluorescence quantitative method. The sequencing was carried out using the Illumina MiSeq platform. Gut microbiota diversity was identified via analyses of alpha and beta diversity, a network analysis, the differentiation of species, examinations of marker species, and functional prediction assessments.

### 2.9. Determination of Fecal SCFAs

The fecal samples were homogenized with water (500 μL), followed by centrifugation to collect the supernatant. Then, 200 μL of supernatant was mixed with 100 μL of 15% phosphoric acid, 20 μL of an internal standard solution (375 μg/mL 4-methylvaleric acid), and 280 μL of ether. The mixture obtained was subjected to centrifugation for 10 min, and the supernatant was collected for a GC-MS analysis using a Thermo Trace 1300 gas chromatograph (Thermo, Waltham, USA) and ISQ 7000 mass spectrometric ((Thermo, Waltham, USA) detection. The GC was fitted with an Agilent HP-INNOWAX capillary column (30 m × 0.25 mm, 0.25 μm) (Agilent, Santa Clara, USA) with helium as a carrier gas at 1 mL/min. The injection of a 1 μL aliquot was administered in split mode (10:1) at an injector temperature of 250 °C. The ion source and MS transfer line temperatures were 300 °C and 250 °C, respectively. The initial temperature was maintained at 90 °C, followed by a programmed increase to 120 °C at a rate of 10 °C/mins, then to 150 °C at a 5 °C/min rate and finally to 250 °C at a rate of 25 °C/min for 2 min. Moreover, 70 eV of energy was applied using the electron impact ionization mode. Mass spectrometry data were acquired in single-ion monitoring mode.

### 2.10. Determination of BAs in Fecal Samples

The fecal samples were ground in a ball mill; mixed with 5 μL of an internal standard solution; then eluted in methanol, precipitated, and centrifuged for a QTRAP 6500+ LC-MS/MS analysis (SCIEX, Darmstadt, Germany). HPLC was performed with a Waters ACQUITY UPLC HSS T3 C_18_ column(100 mm × 2.1 mm i.d.). The elution employed water (containing 0.1% acetic acid and 5 mmol/L ammonium acetate) designated as solvent A and acetonitrile (with 0.01% acetic acid) designated as solvent B. The flow rate was 0.35 mL/min and the elution gradient was as follows: 95% to 60% A in 1 min, then decreased to 50% A in 6 min, decreased to 25% A in 7 min, decreased to 5% A in 14 min, and then ramped back up to 95% A for 2 min. The temperature was 40 °C and the injection volume was 3 μL. The ESI source parameters were set as follows: air curtain gas pressure of 35 psi, ion spray voltage of −4500 V, and temperature of 550 °C.

### 2.11. Western Blotting

Total protein extraction from liver tissue was performed using RIPA buffer (Pioneer Biotechnology, Xi’an, China) and quantified using the BCA assay kit (Pioneer Biotechnology, China). Then, proteins (20 µg) were separated via 10% SDS-PAGE gel electrophoresis, transferred to a polyvinylidene fluoride membrane, blocked with 5% nonfat milk, and incubated overnight at 4 °C with the following primary antibodies: PI3K p85 (1:5000, Proteintech, Shanghai, China), Akt (1:1000, CST, Danvers, MA, USA), p-Akt (1:1000, CST, USA), FGF15 (1:1000, Abcam, Cambridge, UK), FXR/NR1H4 (1:2000, HUABIO, Hangzhou, China) and β-actin (1:10,000, Proteintech, China), and GAPDH (1:10,000, Proteintech, China). The membranes were then incubated with appropriate secondary antibodies (anti-mouse IgG, HRP-linked antibody, 1:10,000; anti-rabbit IgG, HRP-linked antibody, 1:10,000; Proteintech, China) for 2 h at room temperature. The bands were visualized with Enhanced Chemiluminescence (ECL) Plus (Proteintech, China), and their intensity was assessed via Image J 1.37V software.

### 2.12. Statistical Analysis

The data are presented as the mean ± standard deviation (SEM); n denotes the number of mice. All data underwent normality testing via the Shapiro–Wilk test, and equal variance was assessed using the Brown–Forsythe test. For the intergroup statistical comparisons, a one-way ANOVA and Tukey’s multiple comparison test were conducted. The correlations were analyzed using Spearman’s correlation test. For 16S rRNA sequencing data, the Kruskal–Wallis test was employed to identify significant differences across different groups. Here, *p*-values < 0.05 were deemed significant. All analyses were carried out with GraphPad Prism version 10.1.2 software.

## 3. Results

### 3.1. The Chemical Makeup and Quantification of the EC

**Figure 1:**
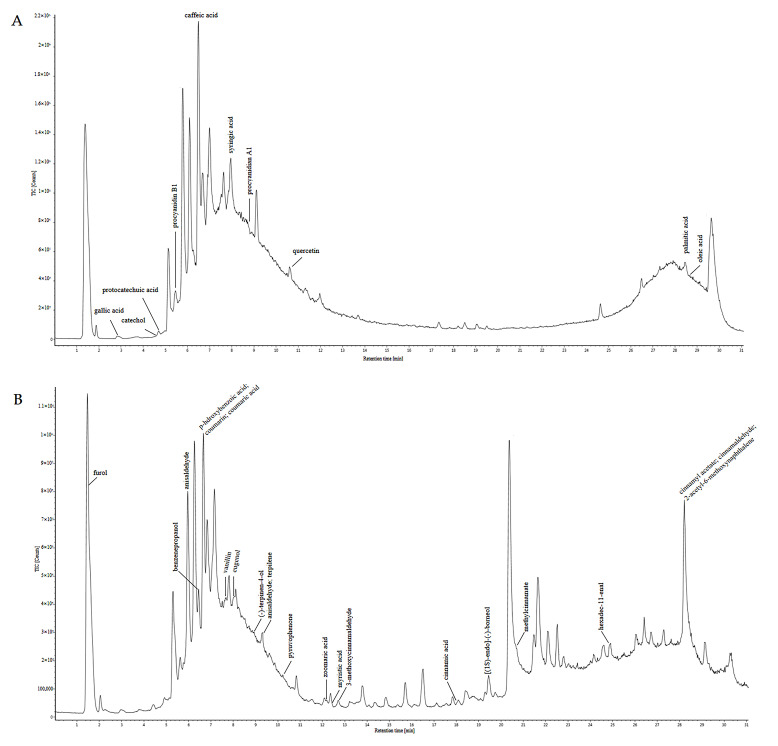
Chemical composition of cinnamon and total ion chromatography in positive ion mode (**A**) and negative ion mode (**B**).

**Figure 2:**
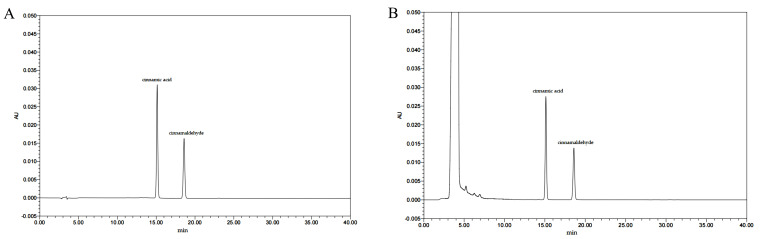
Typical chromatograms of the analyzed components: (**A**) chromatogram of reference materials; (**B**) chromatogram of EC sample.

The examination of the chemical components of the EC was conducted using UPLC-ESI-Q/TOF-MS/MS in both positive and negative ion modes. The total ion chromatogram (TIC) was illustrated in Figure 1. Furthermore, the mass spectral data were processed, analyzed, and matched against the compound database, which revealed 32 chemical components. These components mainly comprise aldehydes such as cinnamaldehyde, polyphenols such as caffeic acid, and phenolic acids such as coumarinic acid. Supplementary Materials S1 provides detailed information on the identified EC components. Figure 2 shows a typical chromatogram. Table 1 summarizes the quantity results of the individual components. Each sample was quantitatively analyzed in triplicate. The quantities of cinnamic acid and cinnamaldehyde were 6.269 ± 0.213 mg/g and 6.133 ± 0.155 mg/g, respectively.

### 3.2. Identification of Active Compounds and Target Discovery

#### 3.2.1. Screening of T2DM-Related Targets Corresponding to EC

**Figure 3:**
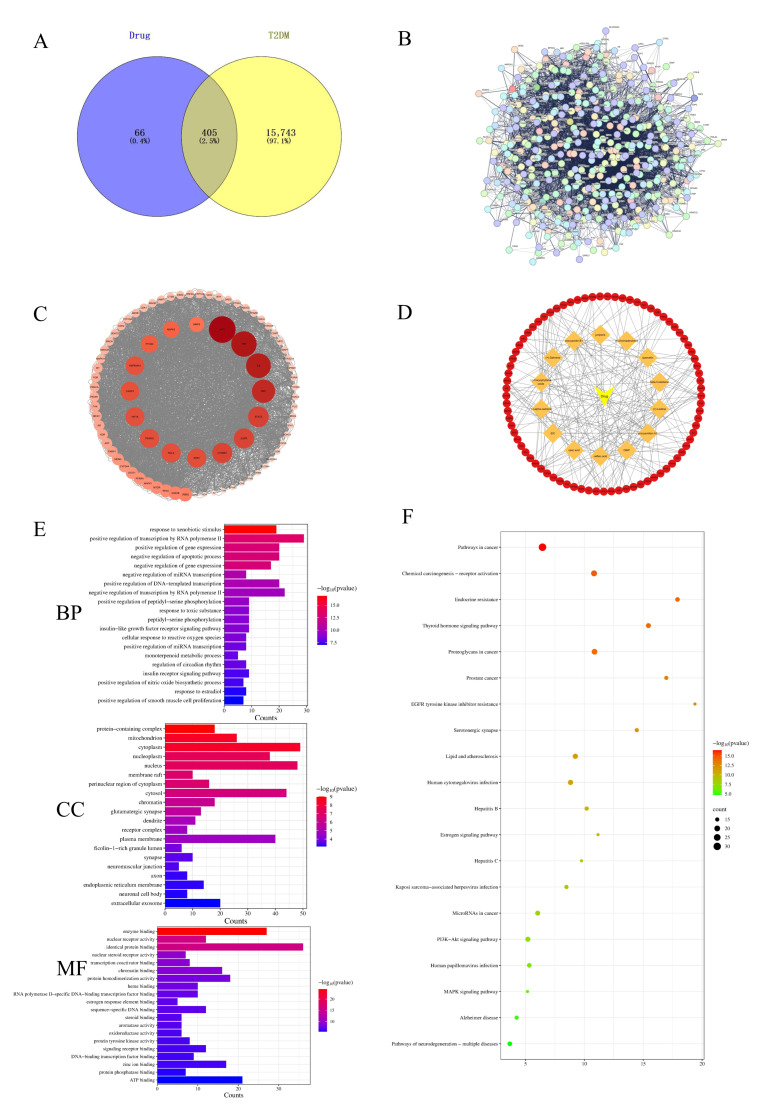
Network pharmacological prediction of EC in the treatment of T2DM: (**A**) Venn diagram of EC targets and T2DM targets; (**B**) protein–protein interaction (PPI) network obtained from the STRING database; (**C**) PPI network of the anti-T2DM targets of EC embellished by Cytoscape; (**D**) ingredient–target network; (**E**) GO enrichment analysis results; (**F**) KEGG enrichment analysis results.

The network pharmacology and UPLC-ESI-Q/TOF-MS/MS analysis revealed 471 targets related to the 14 compounds. The screening process involved searching the TCMSP and SwissTargetPrediction databases. The results were then normalized in UniProt. A total of 16,148 T2DM targets were acquired through a keyword search. Subsequently, the active EC components’ targets were overlapped with T2DM targets via a Venny diagram, which identified 405 EC targets related to the T2DM treatment (Figure 3A).

#### 3.2.2. Analysis of EC’s Multi-Component and Diverse Target Role in Treating T2DM

The PPI network was constructed using STRING. There were 404 nodes and 6252 edges in the PPI network (Figure 3B). Subsequently, the STRING results were imported into Cytoscape software 3.9.1 to evaluate high-interacting protein modules. Nodes indicate target proteins, where darker colors and larger sizes represent higher degree values. In PPI networks, nodes with greater degree values generally assume a more crucial role. Here, 81 targets, including Akt1, TNF, IL6, and PPARG, were identified as the core targets (Figure 3C). To identify the relationships of ingredients and targets, Cytoscape software 3.9.1 was utilized to construct the “ingredient–target interaction” network (Figure 3D). The potential core targets for T2DM are shown in Supplementary Materials Table S2.

#### 3.2.3. Enrichment Analyses of GO and KEGG Pathways

The EC target genes against T2DM were subjected to a GO analysis using the DAVID 6.8 database. The analysis identified 517 biological processes (BPs), 70 cell components (CCs), and 156 molecular functions (MFs) (Figure 3E). The top 20 enriched BP GO terms comprised the response to negative regulation of the apoptotic signaling pathway, insulin-like growth factor receptor signaling pathway, cellular response to reactive oxygen species, and insulin receptor signaling pathway, as well as other biological-process-related terms. Furthermore, the CC terms included the core targets of EC, which were mainly enriched in protein-containing complexes, membrane rafts, and receptor complexes. The enriched MFs of the core EC targets included identical protein binding, protein tyrosine kinase activity, signaling receptor binding, and protein phosphatase binding. To further identify the potential mechanisms, a KEGG pathway enrichment analysis was carried out, which identified 168 related signaling pathways. Figure 3F displays the top 20 significant pathways. Several major pathways associated with T2DM treatment were identified, including lipid and atherosclerosis pathways, the PI3K-Akt signaling pathway, and the MAPK signaling pathway. These pathways may be how EC exerts its anti-T2DM therapeutic effects.

### 3.3. Effects of EC on BW, FBG, OGTT, and Serum Indices

**Figure 4:**
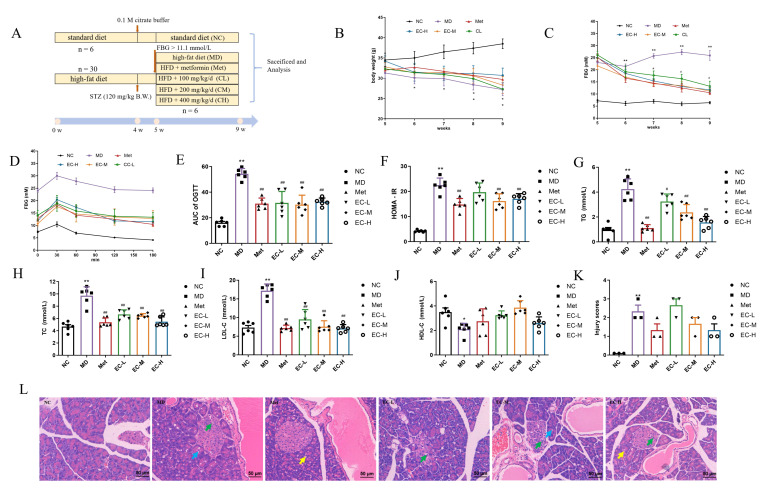
Effects of EC on HFD/STZ-induced diabetic mice: (**A**) experimental design and schedule; (**B**) body weight; (**C**) fasting blood glucose (FBG); (**D**) oral glucose tolerance test (OGTT); (**E**) area under the curve (AUC) of OGTT; (**F**) insulin resistance (HOMA-IR) index; (**G**–**J**) serum biochemical parameters (TG: triglyceride, TC: total cholesterol, HDL-C: high-density lipoprotein cholesterol, LDL-C: low-density lipoprotein cholesterol) and (**K**) the score for degree of lesions; (**L**) H&E staining of the pancreas in each group (scale bar 50 μm). The green arrows represent necrosis of islet cells, the blue arrows represent degeneration of acinar epithelial cells, and the yellow arrows represent autophagic vesicles. Each value is expressed as the mean ± SEM; *n* = 6, * *p* < 0.05, ** *p* < 0.01 vs. NC group; ^#^ *p* < 0.05, ^##^ *p* < 0.01 vs. MD group.

The experimental design is illustrated in Figure 4A. The T2DM model mice had pronounced polyphagia, polyuria, polydipsia, and weight reduction. Relative to the NC group, a substantial decrease in BW and an increase in FBG was observed in the MD mice group (Figure 4B,C). However, the Met and EC treatments effectively reduced the FBG of the diabetic mice compared to the NC group (*p* < 0.0001). The results from the OGTT demonstrated that after intragastric administration of the glucose solution, the blood glucose levels rose significantly within 30 min and gradually declined. Moreover, the highest serum glucose concentrations in the MD mice were about 1.25 times the baseline level, while those in the Met and EC mice were around 1.34–1.72 times the initial levels. Compared to the NC group, the area under the curve (AUC) for the OGTT was highly elevated in the MD group (*p* < 0.0001); however, Figure 4D,E show that these alterations were reversed after the Met and EC interventions, showing statistical significance. Furthermore, the MD group showed increased HOMA-IR levels compared to the NC group. The EC-M and EC-H treatments significantly decreased the HOMA-IR levels (*p* < 0.05, *p* = 0.0023 and 0.0090) (Figure 4F). These findings reveal that EC not only exerts a hypoglycemic effect but also leads to improved glucose tolerance and insulin resistance in T2DM mice.

Diabetes patients have aberrant blood lipid metabolism. Here, it was noted that in comparison to the NC group, the MD group exhibited markedly higher serum concentrations of TC, TG, and LDL-C. The treatments with Met and EC effectively decreased the TC, TG, and LDL-C levels (*p* < 0.05 or *p* < 0.01) (Figure 4G–I). Compared with the MD group, EC increased the HDL-C levels. However, this change did not reach statistical significance (Figure 4J). Figure 4L shows the results of the pancreas histomorphology assessment. The pancreatic pathology was scored based on indicators of islet cell necrosis, acinar epithelial cell degeneration, and autophagic vesicles in acinar cells. The results of the HE staining displayed that necrosis of islet cells and degeneration of acinar epithelial cells were observed in MD mice. However, the lesion scores of the Met and EC-L/M/H groups did not show significant changes compared to those of the MD group (Figure 4K; *p* > 0.05, *p* = 0.1552, 0.9020, 0.4543, and 0.1552, respectively).

### 3.4. EC Inhibited Inflammation and Improved Intestinal Barrier Damage

**Figure 5:**
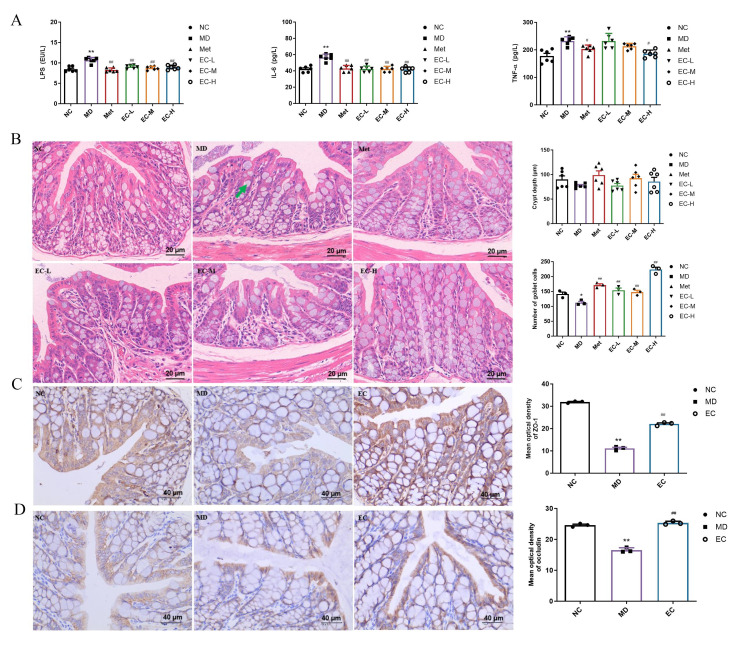
Effects of EC on inflammation and gut barrier: (**A**) the contents of LPS, IL-6, and TNF-α; (**B**) H&E staining of colon tissues; (**C**) ZO-1 and (**D**) occludin immunohistochemical staining of tight-junction proteins associated with the gut barrier:. The green arrow represents goblet cells. Each value is expressed as the mean ± SEM; *n* = 3, * *p* < 0.05, ** *p* < 0.01 vs. NC group; ^#^ *p* < 0.05, ^##^ *p* < 0.01 vs. MD group.

It has been reported that diabetes is often accompanied by gut barrier disruption [17]. LPS is a serum marker for gut barrier damage. Therefore, this study assessed the serum levels of LPS and proinflammatory cytokines (IL-6 and TNF-α). The results showed that compared with the NC group, the levels of LPS, IL-6, and TNF-α in the MD group increased significantly, which were reduced by the EC treatment (*p* < 0.05) (Figure 5A). Our analysis of the colonic histology revealed marked decreases in the quantities of goblet cells within the MD group. Furthermore, the EC-L/M/H group mice indicated effective amelioration of these pathological changes (*p* < 0.05, *p* = 0.0029, 0.0084, and <0.0001, respectively). However, EC did not affect the depths of crypts of intestinal tissues (Figure 5B). ZO-1 and occludin are key components of intestinal tight-junction proteins. The immunohistochemistry revealed that relative to the NC mice, the levels of protein expression for ZO-1 and occludin in the MD mice declined (*p* < 0.05, *p* = 0.0002 and 0.0003); however, the treatment with EC reversed these alterations (*p* < 0.05, *p* < 0.0001 and 0.0002) (Figure 5C,D). Overall, these data suggest that EC can inhibit inflammation and alleviate intestinal barrier damage in T2DM mice.

### 3.5. EC Modulated the Gut Microbiota in T2DM Mice

**Figure 6:**
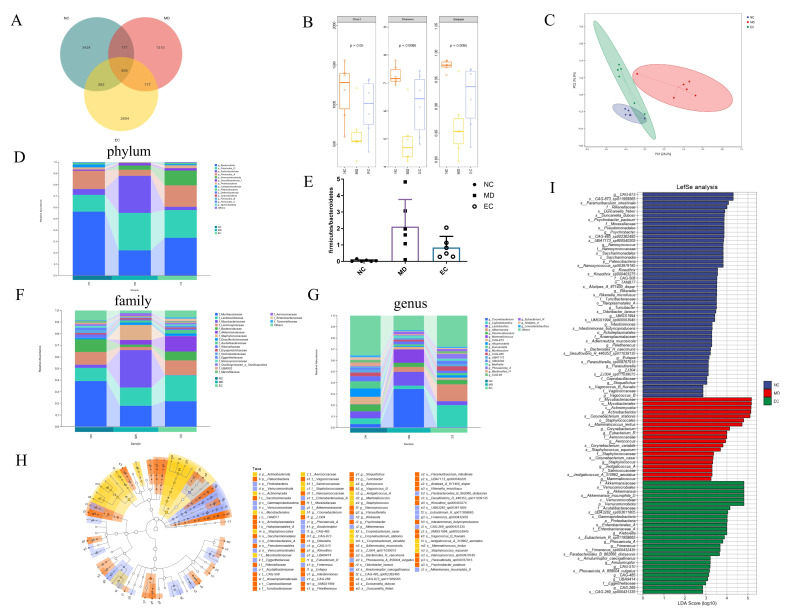
Effect of EC on the gut microbiota in T2DM mice. (**A**) OTU Venn diagram; (**B**) alpha diversity index; (**C**) beta diversity index (Bray–Curtis distance) (ellipses denote 95% confidence intervals); (**D**) relative abundance at phylum level; (**E**) *Firmicutes/Bacteroidetes* (F/B) ratio at the phylum level; (**F**) family and (**G**) genus relative abundance levels; (**H**) taxonomic cladogram and (**I**) histogram from the LEfSe analysis (LDA values were higher than 2). Statistical significance was determined using Kruskal–Wallis test. *n* = 6.

To identify the differences in the gut microbiota of the NC, MD, and EC-H (denoted as EC group in the following sections) groups, 16S rRNA sequencing was carried out. The Venn diagram identified 8683 operational taxonomic units (OTUs) from 3 groups, where the EC group indicated a higher number of OTUs compared to the MD group (Figure 6A). The analysis using the Chao 1, Shannon, and Simpson indices indicated that the alpha diversity was notably greater in the NC group compared to the MD group (*p* < 0.05, *p* = 0.05, *p* = 0.0086, and *p* = 0.0055, Figure 6B). The β-diversity result showed significant differences between the NC and MD groups. However, the EC treatment restructured the microbial composition (Figure 6C). At the phylum level (Figure 6D), the species exhibiting comparatively higher abundance included *Bacteroidota*, *Firmicutes*, and *Actinobacteriota*. The MD mice had a significant reduction in *Bacteroidota* and relative increases in *Firmicutes* and *Actinobacteriota* compared to the NC mice, which was reversed by the EC treatment. Earlier research has shown that the *Firmicutes*-to-*Bacteroidetes* (F/B) ratio is elevated in rat models of T2DM [18]. In this study, compared to the MD group, a remarked reduction in this ratio was observed in the NC group (*p* < 0.05, *p* = 0.0105), whereas the EC groups did not significantly affect this ratio (*p* > 0.05, *p* = 0.0989, Figure 6E). Further, EC markedly enhanced the prevalence of *Verrucomicrobia* across the phylum, class, and order levels; increased the levels of *Muribaculaceae* and *Lactobacillaceae*; and reduced the levels of *Mycobacteriaceae* at the family level. Furthermore, at the genus tier, there was a significant rise in the relative abundance of *Akkermansia* and *Ligilactobacillus* (Figure 6F,G). The LefSe (LDA > 2) analysis was conducted to identify potential biomarkers (Figure 6H,I). In the MD group, there were 20 characteristic bacterial communities, including *Mycobacteriaceae* at the order and family levels; *Actinobacteriota* at the phylum level; and *Corynebacterium*, *Eubacterium*, and *Staphylococcus* at the genus level. In the EC group, 27 characteristic bacterial communities were identified, primarily consisting of *Akkermansiaceae* at the family, genus, and species levels and *Verrucomicrobiales* at the phylum, class, and order levels.

### 3.6. EC Increased the SCFA Content and Affected the Profile of BAs in the Feces of T2DM Mice

**Figure 7:**
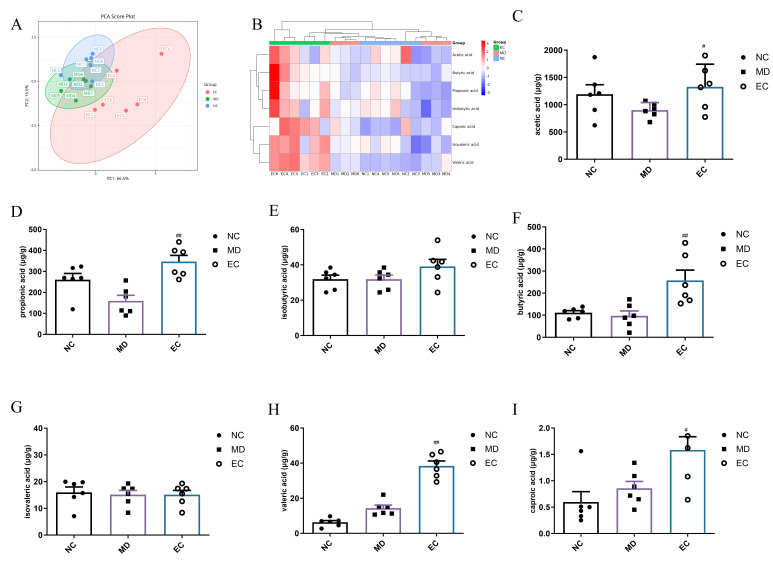
Effect of EC on the SCFAs in T2DM mice: (**A**) PCA plot of fecal SCFAs (ellipses denote 95% confidence intervals); (**B**) heatmap of fecal SCFAs; (**C**–**I**) the levels of acetic acid, propionic acid, isobutyric acid, butyric acid, isovaleric acid, valeric acid, and caproic acid. Each value is expressed as the mean ± SEM; *n* = 6; ^#^ *p* < 0.05, ^##^ *p* < 0.01 with respect to the MD group.

SCFAs and BAs are metabolites produced by the gut, which in turn regulate gut microbiota. This study quantified SCFAs and BAs in mice feces using GC-MS and LC-MS, respectively. The SCFA and bile acid assay validation parameters are shown in Supplementary Materials Tables S3 and S4. Furthermore, the composition and content of SCFAs in the feces of mice were studied using a PCA analysis (Figure 7A). The results showed differences in SCFAs between the EC and MD groups. Moreover, the heatmap analysis showed substantial alterations in SCFA composition and abundance in the EC-treated T2DM mice (Figure 7B). In comparison to the NC group, the concentrations of acetic, propionic, isovaleric, isobutyric, caproic, and valeric acids remained relatively stable between the two groups. Moreover, the EC group exhibited notably higher concentrations of acetic acid (*p* < 0.05, *p* = 0.0493), propionic acid (*p* < 0.01, *p* = 0.0020), butyric acid (*p* < 0.01, *p* = 0.0032), valeric acid (*p* < 0.01, *p* < 0.0001), and caproic acid (*p* < 0.05, *p* = 0.0161) in comparison to the MD group (Figure 7C–I). These results suggested that the modulation of gut microbiota and promotion of SCFAs play important roles in EC-mediated blood glucose regulation.

**Figure 8:**
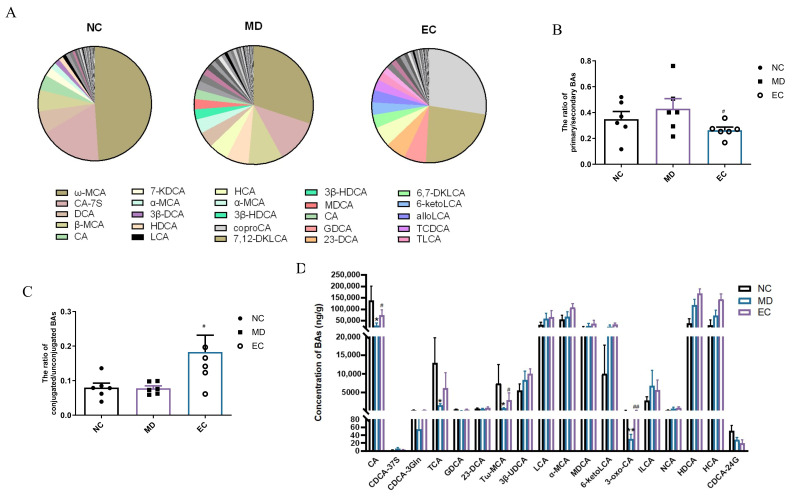
Effect of EC on the BAs in T2DM mice: (**A**) the profiles of BAs in different groups; (**B**) the ratio of primary to secondary BAs; (**C**) the ratio of conjugated to unconjugated BAs; (**D**) primary BA. Each value is expressed as the mean ± SEM; * *p* < 0.05, ** *p* < 0.01 vs. NC group; ^#^ *p* < 0.05, ^##^ *p* < 0.01 vs. MD group.

Figure 8A shows the BA profiles in different groups. The MD mice’s BA profile differed significantly from that of the NC group. Furthermore, the EC group demonstrated a remarkable reversal of altered BA distribution towards that of the NC group, indicating that EC can partially restore the BA profile in T2DM mice. Moreover, the ratio of primary to secondary BAs was greater in the MD group compared to the NC group, which was significantly reversed by the EC treatment, although the difference was not statistically significant (*p* > 0.05, *p* = 0.1213, Figure 8B). Further, no statistical difference was observed in the ratio of conjugated to unconjugated BAs between the NC and MD groups. However, the EC treatment enhanced this ratio, suggesting its potential regulatory role in the BA conjugation status (*p* < 0.05, *p* = 0.0480) (Figure 8C). These alterations were mainly induced by alterations in individual BAs. A total of 18 BAs exhibited variable importance, with projection (VIP) scores > 1 (Figure 8D).

### 3.7. EC Activated Hepatic PI3K/AKT and Colon BA/FXR/FGF15 Signaling Pathways

**Figure 9:**
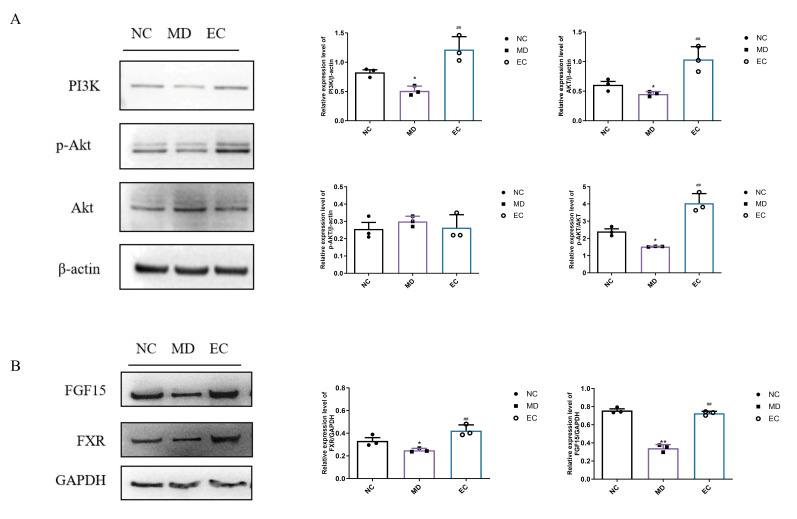
Effect of EC on the (**A**) PI3K/Akt and (**B**) FXR/FGF15 signaling pathways of protein expression. Each value was expressed as the mean ± SEM. * *p* < 0.05, ** *p* < 0.01 vs. NC group, ^##^ *p* < 0.01 vs. MD group.

The PI3K/Akt pathway is released in response to insulin and has been found to modulate glucose homeostasis [19]. Here, the network pharmacology revealed that EC might use the PI3K/Akt signaling pathway to induce anti-T2DM effects. In the MD group, the PI3K and Akt expression was reduced compared to the NC group. However, the EC treatment substantially increased these proteins’ expression (*p* < 0.01, *p* = 0.0046, and *p* = 0.0031, Figure 9A). Although EC does not significantly impact the protein expression of p-Akt, the relative protein expression level of p-Akt/Akt was significantly increased (*p* < 0.01, *p* = 0.0009), indicating that EC improves glucose and lipid metabolism by activating the PI3K/Akt pathway. FXR functions as a nuclear receptor that is activated by bile acids (BAs), predominantly found in both the liver and intestine. This receptor has been shown to influence the production, elimination, and reuptake of bile acids. Following the activation by bile acids, the intestinal FXR enhances the expression of the downstream factor, FGF15. The total protein expression levels of FXR and FGF15 were markedly reduced (*p* < 0.05, *p* = 0.0125, and *p* < 0.0001); however, these reductions were effectively alleviated by the EC treatment (*p* < 0.01, *p* = 0.0008, and *p* < 0.0001) (Figure 9B).

### 3.8. Spearman’s Correlation Analysis

**Figure 10:**
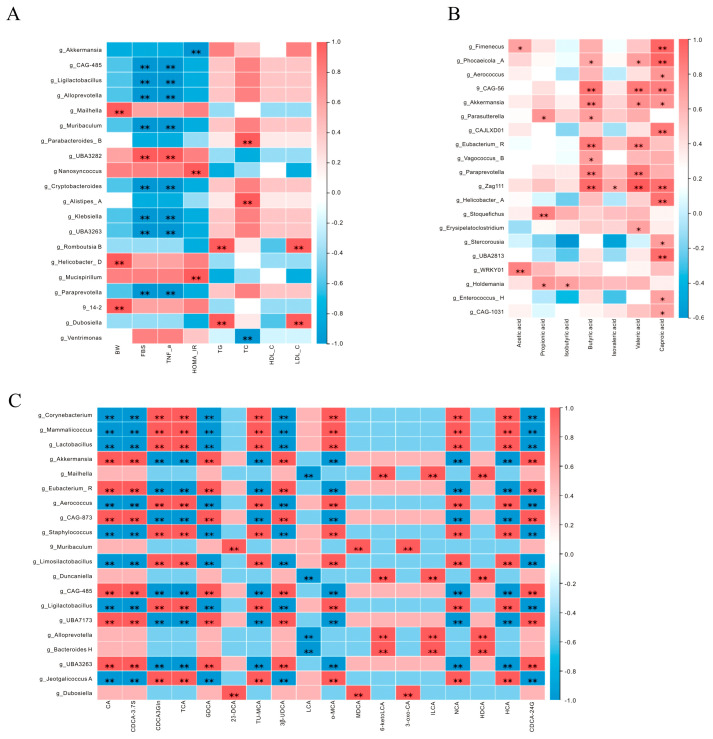
The Spearman analysis between gut microbiota, hypoglycemic effects, SCFAs, and BAs: (**A**) heatmap of the correlation between gut microbiota and hypoglycemic effect; (**B**) heatmap of the correlation between gut microbiota and SCFAs; (**C**) heatmap of the correlation between gut microbiota and BAs; * indicates significant correlations, * *p* < 0.05 and ** *p* < 0.01.

A Spearman correlation analysis was carried out to assess the relationships among the gut microbiota, hypoglycemic effects, and SCFAs. At the genus level, *Akkermansia*, *Ligilactobacillus*, *Alloprevotella*, *Cryptobacteroides*, and *Klebsiella* were negatively associated with FBS, TNF-α, and HOMA-IR. Furthermore, *UBA3282*, *Paraprevotella*, *Alistipes romboutsia*, *Helicobacter*, and *Mucispirillum* were positively associated with glucose- and lipid-metabolism-related indicators (Figure 10A). Similarly, the correlation analysis of gut microbiota and SCFAs showed that *Akkermansia*, *Fimenecus*, *Phocaeicols*, *Parasutterella*, *Eubacterium*, *Paraprevotella*, and *Holdemania* were positively correlated with SCFAs (Figure 10B). Figure 10C illustrates the relationships between gut microbiota and BAs. The alterations in gut microbial abundance directly influenced the levels of primary and secondary BAs. *Akkermansia* was positively associated with cholic acid (CA).

## 4. Discussion

Social and economic development has promoted unhealthy lifestyles, which have consequently increased the incidence of T2DM, making it the most prevalent chronic disease globally. T2DM poses a significant threat to people’s health and lives and greatly elevates the financial strain on healthcare systems worldwide [20]. Several studies have emphasized the efficacy of medicinal herb compounds against T2DM and estimated that about 250,000 herbs can cause reduced glucose production and help combat secondary ailments. However, only 1% have been pharmacologically proven [21].

Cinnamon has been widely used as a medicine and culinary spice for its antipyretic, analgesic, and antiseptic properties. A literature survey spanning the years 2020 to 2022 has demonstrated that cinnamon possesses an insulin-mimetic effect and enhances enzyme activity to modulate glucose metabolism [22]. These beneficial effects of cinnamon have been attributed to its bioactive components, including cinnamaldehyde, polyphenols, and flavonoids. Previous studies proposed that continuous cinnamaldehyde administration for 4 weeks significantly increased insulin and decreased TG and LDL-C levels in T2DM rats [23]. Cinnamon polyphenols achieve hypoglycemic and hypolipidemic effects by repairing pancreatic beta cells, increasing anti-oxidative capacity levels, and alleviating cytotoxicity [24]. Despite evidence indicating that cinnamon has a therapeutic effect on diabetes, its adverse effects and safety should not be ignored. Studies have shown that the safety profile of cinnamon is linked to certain parameters, including FBG, insulin, and aminotransferase levels [25]. Cinnamon in culinary doses (1–6 g), administered as single doses or daily for 2–24 weeks, can exert positive effects on glucose or insulin [26]. However, the presence or absence of beneficial outcomes correlated poorly with the dosage, which may be attributed to confounding variables such as diet and microbiota variability.

The potential molecular mechanisms underlying the anti-T2DM effect of EC were predicted and elucidated using a systematic pharmacological approach. The data revealed that 14 active EC compounds were associated with the anti-T2DM therapeutic effect, highlighting EC’s multi-component and multi-target nature. The screening and prediction process identified 81 essential targets related to T2DM and EC compounds, including Akt1, TNF, IL6, and PPARG. The GO enrichment analysis indicated that EC influenced various biological processes, such as the insulin-like growth factor receptor signaling pathway, the cellular response to reactive oxygen species, and the insulin receptor signaling pathway. Most of these processes were significantly associated with T2DM. The KEGG enrichment analysis showed several T2DM-associated signaling pathways, including the lipid and atherosclerosis pathway, the PI3K-Akt signaling pathway, and the MAPK signaling pathway [27]. The signaling pathway involving PI3K-Akt is essential for liver function and influences the metabolism of glycolipids [28], recognized as a classic diabetes pathway with multiple diabetes-associated targets. PI3K is an active center of most insulin metabolic reactions and can increase Akt kinase activity by stimulating the tyrosine phosphorylation of Akt. The Western blotting analyses showed that EC regulates glucose and lipid metabolism through the PI3K-Akt pathway. However, the predictions of network pharmacology will need to be verify using further functional approaches.

Aberrant glucose levels and lipid metabolism are caused by insulin insufficiency or resistance, the most common metabolic change in T2DM [29]. The predominant clinical manifestations of T2DM are hyperglycemia, hyperlipidemia, elevated OGTT results, hyperinsulinemia, and increased HOMA-IR levels [30]. Here, the T2DM mice indicated hyperglycemia (elevated FBG and AUC levels in the OGTT), insulin resistance (elevated HOMA-IR levels), and dyslipidemia (increased serum TG, TC, and LDL-C levels and decreased HDL-C levels). In the current study, 4 weeks of intragastric EC-L administration did not effect HOMA-IR, whereas EC-H significantly reduced HOMA-IR. With the increase in dosage, the effect of EC in reducing the AUC in the OGTT and the TG levels became more and more significant. The EC-L/M/H dose significantly lowered the TG, TC, and LDL-C levels in the serum but not HDL-C. These results showed potential therapeutic effects in a mouse model of T2DM, as EC can reduce blood sugar and lipid levels, improving glucose tolerance and insulin sensitivity in a dose-dependent manner.

Several studies have linked inflammation to gut barrier integrity, which is substantially related to T2DM [31]. As a key pathogenic factor, microbiota alterations are significantly associated with intestinal permeability and can impair the intestinal barrier integrity [32]. Gut permeability is primarily modulated by specific tight-junction proteins, such as ZO-1 and occludin. Therefore, EC’s effect on intestinal permeability was assessed by investigating inflammatory factor levels, the histopathological changes in colon tissues, and tight-junction protein levels. LPS functions as a crucial structural element found within the outer membrane of Gram-negative bacteria. When the intestinal barrier is disrupted, LPS traverses the gut and enters the bloodstream, triggering an inflammatory reaction [33]. Proinflammatory cytokines such as IL-6 and TNF-α modulate the metabolic processes of glucose and lipid metabolism. Compared to the MD group, the serum LPS and IL-6 levels were substantially decreased after treatment with different doses of EC. In terms of the level of serum TNF-α, the low and medium doses of EC had no obvious effect on it, while the high dose of EC showed a marked reduction. EC treatment did not increase the crypt depth but it increased the quantity of goblet cells in the colon; in particular, the high-dose EC treatment showed a 99.4% rise compared with the MD group. EC also upregulated ZO-1 and occludin protein expression. These findings show that EC could improve the gut barrier function and restore intestinal permeability in a dose-dependent manner.

The gut microbiota affects various physiological and metabolic processes in the host. Studies have identified *Verrucomicrobia* as a beneficial bacterium that is widely distributed in the healthy human gut and can regulate glucose and lipid metabolism [34]. Furthermore, *Akkermansia*, as a probiotic, has been found to be negatively related to T2DM [35]. In this study, it was observed that EC treatment enhanced the regulation of gut microbiota in the mouse model of T2DM. The gut microbiota abundance in the EC mice indicated a recovery trend, with significant alterations in its composition, whereas the MD group had substantially increased abundance levels of *Verrucomicrobia* at the phylum, class, and order levels and of *Akkermansia* at the genus level.

SCFAs, as the main metabolites of the gut microbiota, have been associated with T2DM onset and progression [36]. *Bacteroidetes*, *Firmicutes*, *Actinobacteria*, *Lactobacillales*, and *Verrucomicrobia* can produce SCFAs. It has been observed that diabetes reduces SCFA levels, further exacerbating gut microecological dysregulation [37]. Increasing the production of acetate, propionate, and butyrate acids by the microbiota can alleviate T2DM [38,39]. Acetate acid and butyrate acid increase the energy consumption of mice, while propionic acid increases insulin sensitivity [40]. Here, Spearman’s analysis revealed that microbiota such as *Akkermansia* and *Ligilactobacillus* were linked with EC’s hypoglycemic effects and the SCFA content. It can be concluded that EC reduces T2DM by influencing the makeup of the gut microbiota, mainly through the enhancement of bacteria that produce SCFAs.

Studies have indicated that gut microbiota can modify the BA profile by transforming primary BAs into unconjugated secondary BAs and free BAs via deconjugation, dehydroxylation, oxidation, and isomerization. This phenomenon plays a pivotal role in maintaining gut homeostasis and regulating the development of metabolic diseases [41]. This study found that EC treatment significantly modified the BA profile, where the ratio of primary to secondary BAs decreased, while that of conjugated to unconjugated BAs increased. Furthermore, the Spearman correlation analysis identified that *Akkermansia*, *Lactobacillus*, and *Ligilactobacillus* were significantly associated with primary and secondary BA levels. *Akkermansia* was significantly positively correlated with CA levels. Previous studies reported that lithocholic acid (LCA) and CA are the most potent ligands for FXR [42], a predominant BA receptor. It has been identified as a downstream effector that mediates multiple metabolic processes within BA signaling pathways, thereby regulating glucose homeostasis and insulin resistance. Here, the Western blot analysis indicated that the EC mice had elevated FXR and FGF15 protein expression levels, suggesting that EC may active the BA receptor signaling pathways to regulate glucose balance. However, this study did not provide direct evidence to support the above conclusions, and further validation of cellular pathway activation is required.

The findings of this study provide insights into the mechanisms for the hypoglycemic potential of EC. However, there are still several challenges to overcome in translational research studies of EC. First, the anti-hyperglycemic activity evaluations of EC rely primarily on animal tests, and there is a notable absence of data on its potential toxic effects. Previous studies have shown that coumarin exhibits potential hepatotoxicity [43], while cinnamaldehyde can induce genetic alterations in hepatocytes and disrupt respiratory system homeostasis [44]. However, the current toxicological research on the pharmacokinetic properties of cinnamon extract remains in its infancy. Systematic research into the toxicological impacts or pharmacokinetics of cinnamon extract is a direction worthy of attention, especially its long-term toxicity. Second, T2DM is a chronic metabolic disorder characterized by insulin resistance and relative insulin deficiency. Metformin can improve metabolic insulin resistance, and using metformin alone as a comparator only provides a preliminary evaluation of the hypoglycemic activity of EC. Glucagon-like peptide-1 receptor agonists (GLP-1RAs) potently activate insulin secretion. Therefore, adding GLP-1RAs as a control can better evaluate the hypoglycemic activity of EC. Additionally, natural products may exhibit an additive effect when combined with commercial drugs, which could potentially reduce the therapeutic doses of the latter and minimize the side effects. Hence, there is a need to augment investigations into the antidiabetic effects of EC alone or in combination with commercial oral hypoglycemic agents. Third, we observed that EC increased the abundance of Akkermansia and decreased the Firmicutes/Bacteroidetes ratio in the current study. Furthermore, the relationships among the gut microbiota, metabolism-related indicators, SCFAs, and BAs were analyzed using Spearman correlation analyses. However, the causal relationships between these indicators lack further experimental validation. Therefore, our future research studies will use the microbiota transfer method or antibiotic-induced pseudo-germ-free animal models to verify the interactions between EC, microbiota, metabolic indicators, SCFAs, and bile acids. Fourth, our study has preliminarily revealed through Western blotting analyses that the FXR/FGF15 and PI3K/AKT pathways may serve as potential mechanisms underlying the therapeutic effect of EC against T2DM. However, direct evidence establishing a causal link between signaling pathway activation and EC’s anti-diabetic effect remains lacking. Notably, exploring the interactions between EC and target genes will facilitate an understanding of the anti-hyperglycemic mechanism. Therefore, more experiments should be performed with overexpression and knockdown cells and in knockout animals to reveal the underlying mechanism. Fifth, EC is composed of varying amounts of bioactive phytochemicals, with cinnamaldehyde, cinnamic acid, coumarin, and other substances as its key components, which may largely account for the observed bioactive properties. Pinpointing which specific bioactive constituents are responsible for the anti-diabetic effects would have been a valuable addition. Future studies could compare the anti-diabetic efficacy of individual constituents or their combinations to further clarify the phytochemicals with which EC regulates blood glucose.

## 5. Conclusions

Overall, this research evaluated the effective components and anti-diabetic mechanisms of EC via UPLC-ESI-Q/TOF-MS/MS, network pharmacology, and experimental verification. In total, 32 chemical ingredients were identified in EC, of which 14 components were screened as the main active compounds of EC with anti-T2DM activity. Mechanistically, EC alleviated hyperglycemia, insulin resistance, and lipid metabolism abnormalities by protecting the intestinal barrier, modulating the intestinal microbiota, increasing the content of SCFAs, and regulating the profile of BAs, thereby activating intestinal FXR/FGF15 and hepatic PI3K/AKT signaling pathways. The findings of this study provide a preliminary theoretical basis for the hypoglycemic potential of EC.

## Figures and Tables

**Table 1:** The contents of the analyzed components.

Compounds	Content (mg/g)	Regression Equation	R^2^	Linear Range (μg/mL)
cinnamic acid	6.269 ± 0.213	Y = 72537X + 9229.1	0.9997	1–20
cinnamaldehyde	6.133 ± 0.155	Y = 46534X + 4356.1	0.9998	1–20

## Data Availability

The original contributions presented in this study are included in the article and Supplementary Materials. Further inquiries can be directed to the corresponding authors.

## Supplementary Materials

The following supporting information can be downloaded at https://www.mdpi.com/article/10.3390/cimb47070543/s1.

## Abbreviations

The following abbreviations are used in this manuscript:

T2DM	type 2 diabetes mellitus
EC	ethanol extract of cinnamon
BW	body weight
HFD	high-fat diet
FBG	fasting blood glucose
OGTT	oral glucose tolerance test
HOMA-IR	homeostasis model assessment of insulin resistance
TC	total cholesterol
TG	total triglyceride
LDL-C	low-density lipoprotein cholesterol
HDL-C	high-density lipoprotein cholesterol
TNF-α	tumor necrosis factor-α
SCFAs	short-chain fatty acids
STZ	streptozotocin
MS	mass spectrometry
PPI	protein–protein interaction
GO	Gene Ontology
KEGG	Kyoto Encyclopedia of Genes and Genomes
H&E	Hematoxylin and eosin
BAs	bile acids
CA	cholic acid
FXR	farnesoid X receptor
FGF15	fibroblast growth factor 15
PI3K	phosphatidylinositol 3 kinase
AKT	protein kinase B
ZO-1	zonula occludens-1
