# Supplementary material for: The Antidiabetic Mechanisms of Cinnamon Extract: Insights from Network Pharmacology, Gut Microbiota, and Metabolites
Source: Curr Issues Mol Biol. 2025 Jul 12;47(7):543. doi: 10.3390/cimb47070543 (PMC12293137; doi:10.3390/cimb47070543)
Supplement: Supplementary file 1 [file cimb-47-00543-s001.zip › Table S1. The predicted chemical components of cinnamon ethanol extract identified by UPLC-Q-TOF-MSE analysis.pdf]

Table S1. The predicted chemical components of *cinnamon* ethanol extract identified by UPLC-Q-TOF-MS^{E} analysis

No.	RT (min)	Adducts	MS (m/z)	Fragmentation (m/z)	Mass error (ppm)	Formula	Component name
1	1.54	$[M+H]^+$	97.0273	97.07439	-11.6	$C_5H_4O_2$	furol
2	2.87	$[M-H]^-$	169.0142	125.02415	-0.2	$C_7H_6O_5$	gallic acid
3	4.63	$[M-H]^-$	109.0296	109.0296	0.8	$C_6H_6O_2$	catechol
4	4.69	$[M-H]^-$	153.0223	153.0223	19.5	$C_7H_6O_4$	protocatechuic acid
5	5.46	$[M-H]^-$	577.136	407.07668, 289.07150, 245.08174	1.4	$C_{30}H_{26}O_{12}$	procyanidin B1
6	5.93	$[M+H]^+$	137.059	137.059	-4.9	$C_8H_8O_2$	anisaldehyde
7	6.49	$[M-H]^-$	179.0351	179.0351	0.7	$C_9H_8O_4$	caffeic acid
8	6.56	$[M+K]^+$	217.0982	183.09131	-3.4	$C_{12}H_{18}O$	benzenepropanol
9	6.67	$[M+H]^+$	139.0379	139.03795, 271.05935	-8	$C_7H_6O_3$	p-hdroxybenzoic acid
10	6.67	$[M+H]^+$	165.0539	139.03795	-4.5	$C_9H_8O_3$	coumaric acid
11	6.67	$[M+H]^+$	147.0432	139.03795	-5.5	$C_9H_6O_2$	coumarin
12	7.61	[M-H]-	197.0458	197.04576	1.1	$C_9H_{10}O_5$	syringic acid
13	7.76	$[M+H]^+$	153.0547	123.04309	0.4	$C_8H_8O_3$	vanillin
14	8.08	$[M+H]^+$	165.0907	153.01772	-1.7	$C_{10}H_{12}O_2$	eugenol
15	8.74	$[M-H]^-$	575.1223	231.03154, 243.02999	4.9	$C_{30}H_{24}O_{12}$	procyanidian A1
16	9.12	$[M+Na]^+$	177.127	177.127	11.6	$C_{10}H_{18}O$	(-)-terpinen-4-ol
17	9.3	$[M+H]^+$	137.0588	137.0588	-6.6	$C_8H_8O_2$	anisaldehyde
18	9.3	$[M+Na]^+$	91.0532	91.0532	15.1	$C_{10}H_{16}$	terpilene
19	9.87	$[M+H]^+$	149.0594	115.05407	-2.3	$C_9H_8O_2$	pyruvophenone
20	10.63	$[M-H]^-$	301.0355	151.00366	-3	$C_{15}H_{10}O_7$	quercetin
21	12.2	$[M+Na]^+$	277.2164	277.21643, 192.13789	9.5	$C_{16}H_{30}O_2$	zoomaric acid

No.	RT (min)	Adducts	MS (m/z)	Fragmentation (m/z)	Mass error (ppm)	Formula	Component name
22	12.42	$[M+NH_4]^+$	246.2432	246.24318, 197.03575	16.1	$C_{14}H_{28}O_2$	myristic acid
23	12.71	$[M+H]^+$	163.0749	163.07494	-2.6	$C_{10}H_{10}O_2$	3-methoxycinnamaldehyde
24	18.11	$[M+H]^+$	149.0591	149.05911	-4	$C_9H_8O_2$	cinnamic acid
25	19.46	$[M+Na]^+$	177.1262	161.09565	6.7	$C_{10}H_{18}O$	[(1S)-endo]-(-)-borneol
26	20.7	$[M+H]^+$	163.0755	177.09063, 128.14459, 91.05216	1.1	$C_{10}H_{10}O_2$	methylcinnamate
27	24.89	$[M+Na]^+$	256.2626	256.26387	9.1	$C_{16}H_{30}O$	hexadec-11-enal
28	28.23	$[M+H]^+$	177.0904	134.06733	-3.6	$C_{11}H_{12}O_2$	cinnamyl acetate
29	28.23	$[M+H]^+$	133.0637	133.0637	-7.9	C_9H_8O	cinnamaldehyde
30	28.23	$[M+NH_4]^+$	118.0764	161.09530, 177.09065	5.8	$C_{13}H_{12}O_2$	2-acetyl-6-methoxynaphthalene
31	28.45	$[M-H]^-$	255.233	116.92855	0	$C_{16}H_{32}O_2$	palmitic acid
32	28.5	$[M-H]^-$	281.2485	255.23296, 116.92855	-0.2	$C_{18}H_{34}O_2$	oleic acid
