# Supplementary material for: The Antidiabetic Mechanisms of Cinnamon Extract: Insights from Network Pharmacology, Gut Microbiota, and Metabolites
Source: Curr Issues Mol Biol. 2025 Jul 12;47(7):543. doi: 10.3390/cimb47070543 (PMC12293137; doi:10.3390/cimb47070543)
Supplement: Supplementary file 1 [file cimb-47-00543-s001.zip › Table S2. Potential core targets for T2DM.pdf]

Table S2. Potential core targets for T2DM

No.	Target name	Gene name
1	Serine/threonine-protein kinase AKT	AKT1 *
2	TNF-alpha	TNF
3	Interleukin-6	IL6
4	Tyrosine-protein kinase SRC	SRC
5	Signal transducer and activator of transcription 3	STAT3
6	Epidermal growth factor receptor erbB1	EGFR
7	Axin1/beta-catenin	CTNNB1
8	Estrogen receptor alpha	ESR1
9	Apoptosis regulator Bcl-2	BCL2
10	Peroxisome proliferator-activated receptor gamma	PPARG
11	Hypoxia-inducible factor 1 alpha	HIF1A
12	Caspase-3	CASP3
13	Heat shock protein HSP 90-alpha	HSP90AA1
14	Cyclooxygenase-2	PTGS2
15	MAP kinase ERK1	MAPK3
16	Matrix metalloproteinase 9	MMP9
17	Receptor protein-tyrosine kinase erbB-2	ERBB2
18	Glycogen synthase kinase-3 beta	GSK3B
19	cAMP-dependent protein kinase alpha-catalytic subunit	PRKACA
20	Serine/threonine-protein kinase mTOR	MTOR
21	MAP kinase ERK2	MAPK1
22	Peroxisome proliferator-activated receptor alpha	PPARA
23	Signal transducer and activator of transcription 1-alpha/beta	STAT1
24	p53-binding protein Mdm-2	MDM2
25	Cytochrome P450 3A4	CYP3A4
26	Poly [ADP-ribose] polymerase-1	PARP1
27	Vascular endothelial growth factor receptor 2	KDR

28	Beta amyloid A4 protein	APP
29	Androgen Receptor (by homology)	AR
30	Glucocorticoid receptor	NR3C1
31	Tyrosine-protein kinase FYN	FYN
32	PI3-kinase p110-alpha/p85-alpha	PIK3R1
33	Protein kinase C alpha	PRKCA
34	Stem cell growth factor receptor	KIT
35	Progesterone receptor	PGR
36	HMG-CoA reductase	HMGCR
37	MAP kinase p38 alpha	MAPK14
38	Monoamine oxidase A	MAOA
39	Histone deacetylase 2	HDAC2
40	Cyclin-dependent kinase 2	CDK2
41	P-glycoprotein 1	ABCB1
42	Cyclin-dependent kinase 1	CDK1
43	Cytochrome P450 19A1	CYP19A1
44	DNA (cytosine-5)-methyltransferase 1	DNMT1
45	Monoamine oxidase B	MAOB
46	Hepatocyte nuclear factor 4-alpha	HNF4A
47	Cytochrome P450 1A2	CYP1A2
48	Cathepsin (B and K)	CTSB
49	Estrogen receptor beta	ESR2
50	Glucagon	GCG
51	Tyrosine-protein kinase BTK	BTK
52	Dopamine D2 receptor	DRD2
53	ATP-binding cassette sub-family G member 2	ABCG2
54	Cyclooxygenase-1	PTGS1
55	Cytochrome P450 2C9	CYP2C9
56	Catechol O-methyltransferase (by homology)	COMT

57	Alpha-synuclein	SNCA
58	Peroxisome proliferator-activated receptor delta	PPARD
59	Myeloperoxidase	MPO
60	Microtubule-associated protein tau	MAPT
61	Cytochrome P450 2C19	CYP2C19
62	Mu opioid receptor	OPRM1
63	Retinoid X receptor alpha	RXRA
64	Cytochrome P450 2D6	CYP2D6
65	Bromodomain-containing protein 4	BRD4
66	Histone deacetylase 3	HDAC3
67	Plasminogen (by homology)	PLG
68	DNA topoisomerase II alpha	TOP2A
69	Isocitrate dehydrogenase [NADP] cytoplasmic	IDH1
70	Histone deacetylase 4	HDAC4
71	Glutathione reductase	GSR
72	Serotonin transporter	SLC6A4
73	Cytosolic phospholipase A2	PLA2G4A
74	Metabotropic glutamate receptor 5	GRM5
75	Thymidylate synthase	TYMS
76	Acetylcholinesterase	ACHE
77	Epoxide hydratase	EPHX2
78	Transthyretin	TTR
79	Thrombin	F2
80	Xanthine dehydrogenase	XDH
81	Glucose-6-phosphate 1-dehydrogenase	G6PD

* denoted validated targets
