# Supplementary material for: The Antidiabetic Mechanisms of Cinnamon Extract: Insights from Network Pharmacology, Gut Microbiota, and Metabolites
Source: Curr Issues Mol Biol. 2025 Jul 12;47(7):543. doi: 10.3390/cimb47070543 (PMC12293137; doi:10.3390/cimb47070543)
Supplement: Supplementary file 1 [file cimb-47-00543-s001.zip › Table S3. The methodological validation results of SCFAs.pdf]

Table S3. The methodological validation results of SCFAs

SCFAs	Regression equation	r	Linear range (μg/mL)	LOQ (μg/mL)	Recovery rates		
					IS (20μg/mL)	IS (50μg/mL)	IS (200μg/mL)
Acetic acid	Y = 0.002972x + 0.002902	0.9964	0.02 - 500.0	0.02	80.27	102.99	89.73
Propionic acid	Y = 0.005117x + 0.001112	0.9956	0.02 - 500.0	0.02	80.79	97.26	92.02
Isobutyric acid	Y = 0.007775x + 0.0001566	0.9936	0.02 - 500.0	0.02	91.28	105.66	99.51
Butyric acid	Y = 0.01607x + 0.0002845	0.9941	0.02 - 500.0	0.02	88.01	105.69	106.67
Isovaleric acid	Y = 0.01851x + 0.0007127	0.9912	0.02 - 500.0	0.02	100.11	108.27	112.18
Valeric acid	Y = 0.01927x + 3.452e-05	0.9902	0.02 - 500.0	0.02	100.33	110.23	108.21
Caproic acid	Y = 0.06957x + 0.002907	0.9905	0.02 - 500.0	0.02	83.4	89.75	87.11

LOQ: limit of quantification, IS: internal standards. The recovery rates were determined by adding IS (20, 50, and 200μg/mL) to measure the spiked recovery rates.
