# Supplementary material for: The Antidiabetic Mechanisms of Cinnamon Extract: Insights from Network Pharmacology, Gut Microbiota, and Metabolites
Source: Curr Issues Mol Biol. 2025 Jul 12;47(7):543. doi: 10.3390/cimb47070543 (PMC12293137; doi:10.3390/cimb47070543)
Supplement: Supplementary file 1 [file cimb-47-00543-s001.zip › Table S4. The methodological validation results of BAs.pdf]

Table S4. The methodological validation results of BAs

BAs	Regression equation	r	Linear range (ng/mL)	LLOQ (ng/mL)	ULOQ (ng/mL)	Recovery rates (%)
CA-7S	$y = 0.01393\ x + 6.79045\times10^{-4}$	0.9998	4-4000	4	4000	108.19
3-oxo-DCA	$y = 0.16151\ x + 0.00177$	0.9974	1-400	1	400	55.19
β-MCA	$y = 0.02613\ x + 0.00564$	0.9986	4-4000	4	4000	114.37
7-KDCA	$y = 0.00564\ x + 9.79183\times10^{-4}$	0.9995	2-4000	2	4000	93.10
HDCA	$y = 0.00483\ x + 0.00100$	0.9957	10-4000	10	4000	101.23
GLCA	$y = 0.03468\ x + -7.06684\times10^{-4}$	0.9975	10-4000	10	4000	84.32
GCDCA	$y = 0.03263\ x + 0.00643$	0.9983	4-4000	4	4000	99.08
GUDCA	$y = 0.01482\ x + 0.00964$	0.9979	20-4000	20	4000	83.03
GCA	$y = 0.00757\ x + 0.00287$	0.9992	2-4000	2	4000	114.09
GDCA	$y = 0.03199\ x + 0.00246$	0.9994	20-4000	20	4000	103.21
TUDCA	$y = 0.01842\ x + 0.00453$	0.9991	10-4000	10	4000	87.42
THDCA	$y = 0.00955\ x + -2.13688\times10^{-4}$	0.9988	4-2000	4	2000	99.53
GCDCA-3S	$y = 0.06275\ x + 8.91107\times10^{-4}$	0.9991	2-2000	2	2000	104.06
TLCA	$y = 0.13660\ x + -0.00813$	0.9995	4-4000	4	4000	96.88
Tω-MCA	$y = 0.30016\ x + 0.27596$	0.9990	2-4000	2	4000	73.49
TDCA	$y = 0.00105\ x + -4.28424\times10^{-5}$	0.9988	10-4000	10	4000	104.23
TCA	$y = 0.04967\ x + 0.00801$	0.9997	10-4000	10	4000	86.43
Tα-MCA	$y = 0.05777\ x + 0.01055$	0.9988	4-4000	4	4000	95.27
TCDCA	$y = 0.00266\ x + 0.00106$	0.9964	4-4000	4	4000	109.68
CDCA	$y = 0.01874\ x + 0.00826$	0.9995	2-4000	2	4000	103.76
12-oxo-CDCA	$y = 0.02126\ x + 0.01856$	0.9988	2-2000	2	2000	119.10
UDCA	$y = 0.03280\ x + 0.02355$	0.9989	2-2000	2	2000	85.08

6-ketoLCA	y = 0.02688 x + 0.00125	0.9990	4-4000	4	4000	98.72
7-KLCA	y = 0.01671 x + 0.00218	0.9990	4-4000	4	4000	91.02
TCA-3S	y = 0.17992 x + 0.00478	0.9990	1-1000	1	1000	87.82
DCA	y = 0.00308 x + 7.86946×10^{-4}	0.9985	4-4000	4	4000	90.03
12-KLCA	y = 0.04510 x + 0.01852	0.9994	4-1000	4	1000	83.74
3β-HDCA	y = 0.02471 x + 0.00138	0.9997	4-1000	4	1000	99.74
LCA	y = 0.02632 x + 0.03939	0.9989	1-2000	1	2000	104.24
TLCA-3S	y = 0.01274 x + 0.00685	0.9938	10-4000	10	4000	103.64
GUDCA-3S	y = 0.09175 x + 0.00338	0.9996	1-1000	1	1000	90.22
βGCA	y = 0.03420 x + 0.00317	0.9983	4-2000	4	2000	113.26
DLCA	y = 0.07946 x + -0.11001	0.9951	5-400	5	400	23.99
IALCA	y = 0.01954 x + 0.01070	0.9985	2-2000	2	2000	110.11
ILCA	y = 0.03012 x + 0.02109	0.9981	2-4000	2	4000	104.88
alloLCA	y = 0.01346 x + 6.05017×10^{-4}	0.9994	4-4000	4	4000	94.54
23-DCA	y = 0.01885 x + 0.00100	0.9997	2-1000	2	1000	100.16
MDCA	y = 0.02372 x + 0.00332	0.9987	2-4000	2	4000	108.20
3β-UDCA	y = 0.02551 x + 0.00353	0.9964	1-4000	1	4000	108.98
isoCDCA	y = 0.01139 x + 2.88559×10^{-4}	0.9996	4-4000	4	4000	90.00
3β-DCA	y = 0.01074 x + 0.00173	0.9991	1-4000	1	4000	96.72
IDCA	y = 0.00281 x + 3.38809×10^{-4}	0.9994	2-4000	2	4000	83.24
NCA	y = 0.00192 x + 0.00113	0.9963	4-4000	4	4000	110.65
DHCA	y = 0.01858 x + -3.56592×10^{-6}	0.9997	2-4000	2	4000	111.72
7,12-DKLCA	y = 6.75099e-4 x + 3.51976×10^{-4}	0.9959	10-4000	10	4000	108.29
6,7-DKLCA	y = 0.00494 x + 6.01286×10^{-4}	0.9993	1-4000	1	4000	100.76
3-oxo-CA	y = 0.06835 x + -0.48941	0.9980	1-4000	1	4000	11.69
UCA	y = 0.00214 x + 1.48643×10^{-4}	0.9994	10-4000	10	4000	106.71

ω-MCA	$y = 1.57497e-4\ x + 1.38527\times10^{-4}$	0.9994	20-4000	20	4000	127.54
3β-CA	$y = 0.00616\ x + 0.00593$	0.9972	10-4000	10	4000	97.73
α-MCA	$y = 0.02076\ x + 5.90475\times10^{-4}$	0.9993	2-4000	2	4000	31.39
HCA	$y = 0.00929\ x + 0.00430$	0.9983	4-4000	4	4000	115.57
CA	$y = 0.00245\ x + 0.00152$	0.9973	4-4000	4	4000	103.90
LCA-3S	$y = 0.02256\ x + 3.18397\times10^{-4}$	0.9978	4-4000	4	4000	108.90
GDHCA	$y = 0.00709\ x + 0.00604$	0.9921	4-4000	4	4000	83.27
GHCA	$y = 0.00923\ x + 0.00588$	0.9960	10-4000	10	4000	81.35
TDHCA	$y = 9.38847e-4\ x + 9.53343\times10^{-5}$	0.9938	20-4000	20	4000	91.36
GLCA-3S	$y = 0.00462\ x + 9.14814\times10^{-4}$	0.9965	4-4000	4	4000	111.80
Tβ-MCA	$y = 0.04735\ x + 0.01240$	0.9994	4-4000	4	4000	105.40
THCA	$y = 0.05056\ x + 0.03352$	0.9978	10-4000	10	4000	83.03
CDCA-3Gln	$y = 0.03205\ x + 0.02263$	0.9981	2-4000	2	4000	100.79
CA-3S	$y = 0.05777\ x + 9.73985\times10^{-4}$	0.9991	2-4000	2	4000	96.97
CDCA-3S	$y = 0.09404\ x + 0.00746$	0.9990	2-4000	2	4000	93.44
DCA-3-O-S	$y = 0.33611\ x + 0.24212$	0.9964	2-400	2	400	82.79
ACA	$y = 0.00786\ x + 5.18524\times10^{-4}$	0.9892	4-4000	4	4000	115.55
5-isoLCA	$y = 0.01920\ x + 0.00223$	0.9989	2-4000	2	4000	115.43
3-oxoCDCA	$y = 7.57640e-4\ x + -0.00231$	0.9919	40-4000	40	4000	38.25
CA-3G	$y = 5.62922e-4\ x + 7.85634\times10^{-4}$	0.9988	40-4000	40	4000	100.08
12-DHCA	$y = 0.00107\ x + 0.00158$	0.9991	40-4000	40	4000	101.79
dioxo-CDCA	$y = 0.00109\ x + 3.43640\times10^{-4}$	0.9980	40-4000	40	4000	97.09
CDCA-24G	$y = 0.00342\ x + 0.00855$	0.9975	20-4000	20	4000	97.73
11-LCA	$y = 0.01642\ x + 0.00250$	0.9985	2-4000	2	4000	102.81
apoCA	$y = 0.00137\ x + 6.60639\times10^{-4}$	0.9989	4-4000	4	4000	84.37
UDCA-3S	$y = 0.05733\ x + 0.00290$	0.9995	2-4000	2	4000	98.60

dioxo-HDCA	y = 2.73894e-4 x + 2.06516×10^{-4}	0.9994	40-4000	40	4000	96.69
TUDCA-3S	y = 8.66229e-4 x + 2.70614×10^{-4}	0.9969	10-4000	10	4000	55.88
LCA-3G	y = 0.04960 x + 0.00221	0.9988	2-2000	2	2000	100.98
coproCA	y = 0.04821 x + 0.00677	0.9990	1-2000	1	2000	105.20
TUCA	y = 0.75264 x + 0.07463	0.9972	0.4-400	0.4	400	76.26
CDCA-3,7S	y = 0.05113 x + 0.02040	0.9985	2-2000	2	2000	108.88
DCA-3,12S	y = 0.06162 x + 0.00249	0.9987	1-2000	1	2000	107.08

LLOQ: lower limit of quantitation, ULOQ: upper limit of quantitation.
